# Targeting senescence with 5-methyltetrahydrofolate: a novel strategy to attenuate periodontitis

**DOI:** 10.3389/fnut.2026.1851692

**Published:** 2026-08-04

**Authors:** Lisa Yang, Shusen Zhang, Zeyu Zhang, Yuzhen Wang, Huan Zhou, Yuchao Li, Jianfei Tang, Ruoyan Cao

**Affiliations:** 1 School and Hospital of Stomatology, Wenzhou Medical University, Wenzhou, Zhejiang, China; 2 Department of Stomatology, Hunan University of Medicine, Huaihua, Hunan, China; 3 Department of Stomatology, Taizhou Hospital of Zhejiang Province Affiliated to Wenzhou Medical University, Linhai, Zhejiang, China; 4 Key Laboratory of Shaanxi Province for Craniofacial Precision Medicine Research, College of Stomatology, Xi’an Jiaotong University, Xi’an, China; 5 Liaoning Provincial Key Laboratory of Oral Diseases, Department of Periodontics, School and Hospital of Stomatology, China Medical University, Shenyang, China; 6 Hunan Key Laboratory of Oral Health Research, Xiangya School of Stomatology, Xiangya Stomatological Hospital, Central South University, Changsha, Hunan, China

**Keywords:** 5-MTHF, biological aging, NHANES, periodontitis, senescence

## Abstract

**Introduction:**

5-methyltetrahydrofolate (5-MTHF), the biologically active form of folate, possesses well-characterized anti-inflammatory and antioxidant properties that are closely implicated in the regulation of cellular senescence. Accordingly, this study aimed to investigate whether 5-MTHF attenuates periodontitis by mitigating cellular senescence.

**Methods:**

We performed a cross-sectional analysis using data from the National Health and Nutrition Examination Survey (NHANES) 2011-2014 to evaluate the association between serum 5-MTHF levels and periodontitis. Weighted multivariable regression models were applied, and exploratory mediation analysis assessed the contribution of biological aging. In vitro experiments examined the anti-senescence effect of 5-MTHF on lipopolysaccharide (LPS)-induced human gingival fibroblasts (HGFs). An in vivo mouse model of experimental periodontitis was used to evaluate alveolar bone loss.

**Results:**

Among 6,245 adults, higher serum 5-MTHF levels were significantly associated with lower odds of periodontitis, reduced clinical attachment loss, and shallower probing depth. Biological aging mediated 5.5%–17.3% of these associations. Mechanistically, 5-MTHF treatment attenuated LPS-induced cellular senescence in HGFs, as shown by decreased SA-β-gal activity, downregulation of p16, p21, and senescence-associated secretory phenotype genes. Correspondingly, local administration of 5-MTHF significantly alleviated alveolar bone resorption in mice with periodontitis.

**Discussion:**

5-MTHF is associated with improved periodontal health, partly through attenuating cellular senescence. Our findings suggest that targeting senescence with 5-MTHF may represent a novel mechanism-based strategy for preventing and treating periodontitis.

## Introduction

Periodontitis, a chronic inflammatory disease triggered by microbial biofilms, affects more than 50% of adults worldwide (1). It is strongly associated with various systemic diseases, such as diabetes and cardiovascular disease, substantially reducing quality of life and contributing significantly to the global disease burden. Growing evidence indicates an important link between nutritional status and periodontal health. Building on our previous findings, which revealed protective effects of dietary antioxidants and retinol intake on periodontal health (2, 3), further comprehensive investigation into the relationship between nutrition and periodontitis is warranted.

Folate, a water-soluble B9 vitamin abundant in grains and leafy greens, serves as an essential cofactor in one-carbon metabolism and is critical for DNA synthesis and cellular division (4). Upon intestinal absorption, dietary folate is metabolized to its principal circulating form and biologically active form, 5-methyltetrahydrofolate (5-MTHF). Unlike synthetic folic acid, which is biologically inactive and must undergo enzymatic reduction and methylation to become functional, 5-MTHF is directly utilizable by the body. Tetrahydrofolate (THF), an intermediate active form, is highly unstable and prone to oxidation, making it unsuitable for supplementation. This metabolic advantage gives 5-MTHF superior bioavailability and makes it unaffected by common genetic polymorphisms.

Beyond its fundamental metabolic functions, 5-MTHF exhibits pleiotropic bioactivities. It has been shown to suppress NF-κB activation and subsequent pro-inflammatory cytokine production, including IL-1β, IL-6, and TNF-α, while also mitigating oxidative stress through scavenging reactive oxygen species (ROS) and strengthening endogenous antioxidant defenses (5–7). As inflammation and oxidative stress are established drivers of periodontal tissue destruction, 5-MTHF emerges as a compelling candidate for modulating periodontitis pathology.

Furthermore, inflammation and oxidative stress are established contributors to the aging process. Aging is a multifaceted phenomenon characterized by progressive physiological decline and increased susceptibility to age-related diseases, including periodontitis. Cross-sectional data from the National Health and Nutrition Examination Survey (NHANES) have revealed associations between accelerated biological aging, α-Klotho depletion, telomere length attrition, and periodontitis (8–10). Animal studies further corroborate these findings, indicating that aging promotes osteoclastogenesis and alveolar bone resorption (11). This convergence of pathways suggests that biological aging may serve as a critical mechanistic link between 5-MTHF status and periodontal pathology.

At the cellular level, a key manifestation of biological aging is cellular senescence, a state of irreversible growth arrest accompanied by a pro-inflammatory secretome. We therefore hypothesize that targeting cellular senescence with 5-MTHF could be a novel strategy to mitigate periodontitis. To explore this hypothesis at the population level, we utilized data from the NHANES. As a nationally representative, multi-ethnic population-based survey, NHANES substantially reduces selection bias and affords far greater generalizability than small, regionally constrained clinical study samples. Its rigorously standardized clinical examination protocols and quality-controlled laboratory assays minimize measurement error and improve result reproducibility. Furthermore, its large sample size provides sufficient statistical power to detect modest associations and adjust for a broad range of confounding variables, strengthening both the internal and external validity of population-level findings. Thus, we employed a translational approach, integrating analyses of a national population survey (NHANES) with *in vitro* and *in vivo* models to investigate whether 5-MTHF attenuates periodontitis, in part, by alleviating senescence-associated mechanisms.

## Materials and methods

### Study population

This cross-sectional investigation employed three cycles of survey data sourced from NHANES conducted between 2011 and 2014. Data from NHANES were collected through interviews, alongside laboratory and physical assessments, with all participants furnishing written informed consent. In this research, individuals who did not undergo a comprehensive full-mouth periodontal examination (FMPE) and those devoid of data on serum 5- MTHF and biological aging were omitted.

### Exposure variable

5-MTHF was measured by isotope-dilution high performance liquid chromatography coupled to tandem mass spectrometry (LC-MS/MS) (Fazili and Pfeiffer, 2013). The assay is performed by combining specimen (275 μL serum) with ammonium formate buffer and an internal standard mixture. Sample extraction and clean-up is performed by automated solid phase extraction (SPE) using 96-well phenyl SPE plates and takes ∼5 h for a 96-well plate. Folate forms are separated within 6 min using isocratic mobile phase conditions and measured by LC-MS/MS. Quantitation is based on peak area ratios interpolated against a five-point aqueous calibration curve.

### Outcome variable

Periodontal assessments adhered to the FMPE protocol, recording attachment loss (AL) and probing pocket depth (PPD) at six specific sites on each tooth, with the exception of third molars. Periodontitis classification was defined according to CDC/AAP criteria (Supplementary Table 1). In addition, the average values for AL and PPD were utilized to further assess the periodontal condition.

### Mediator

Biological aging served as the mediator, assessed using two established metrics: Phenotypic Age (PhenoAge) and the Klemera-Doubal method biological age (KDM-BA). PhenoAge employs a multivariate mortality hazard model that incorporates nine clinical biomarkers, which allows for a more accurate evaluation of the aging process and vulnerability to age-related diseases compared to relying solely on chronological age (12). Conversely, KDM-BA is derived from a regression analysis that compares aging biomarkers with chronological age in population references. An elevated KDM in relation to chronological age signifies a heightened risk for age-related diseases and mortality, whereas lower KDM values suggest a more robust aging process (13). Both metrics were calculated utilizing the BioAge package in R. The acceleration of biological aging was determined by calculating the residuals from a regression of biological age against chronological age.

### Covariates

The covariates available in the NHANES database were collected based on previous studies, and include the following variables: age (≤60 or >60 years), sex (male or female), race (non-Hispanic White, non-Hispanic Black, Mexican American, or others), education level (<high school, high school, or >high school), annual household income (<US$20,000 or >US$20,000), marital status (never married, married/living as married, or separated/divorced/widowed), smoking status (non-smokers or smokers), recreational activity (no, moderate, vigorous, or both), work activity (no, moderate, vigorous, or both), obesity (non-obese: BMI < 30 or obese: BMI ≥ 30), healthy diet index-2015 (HEI-2015), diabetes mellitus, cardiovascular disease (CVD), and hypertension.

### Cell culture and drug administration

Human gingival fibroblasts (HGFs) were isolated from healthy donors undergoing crown-lengthening surgery, following approval from the Medical Ethics Committee of China Medical University and Stomatology Hospital (2017 Scientific Ethics Review No. 5). Written informed consent was obtained from all participants prior to sample collection. The HGFs were cultured in α-minimum essential medium (α-MEM, Gibco, United States), which was supplemented with 10% fetal bovine serum (FBS, Gibco, United States). The cells were maintained at 37 °C in an atmosphere containing 5% CO_2_. For experimental purposes, HGFs were utilized between passages 3 and 8.

For drug treatment, HGFs were first incubated with 10 μM or 20 μM 5-MTHF (MedChemExpress, United States) for 2 h. Subsequently, the cells were stimulated with 2 μg/mL LPS from Escherichia coli (Cat. No. L2880, ≥97%, Sigma, Germany). After stimulation, all cell cultures were continued in α-MEM supplemented with 10% FBS for a duration of 24 h.

### Senescence-associated β-galactosidase (SA-β-gal) staining

Cellular senescence was evaluated by senescence-associated β-galactosidase (SA-β-gal) activity assay using a commercial kit (Beyotime Institute of Biotechnology, China) according to the manufacturer’s instructions. Briefly, cells were fixed for 15 min, washed with PBS, and then incubated with the staining solution at 37 °C for 16 h. SA-β-gal-positive cells were visualized and imaged under an inverted light microscope (Zeiss Axio).

### Real-time quantitative PCR (rT-qPCR)

Total RNA was extracted using the RNA-quick Purification Kit (ESscience Biotech, China) according to the manufacturer’s instructions. Subsequently, 1 μg of total RNA was reverse-transcribed into cDNA using HiScript III RT SuperMix (Vazyme, China). The primers designated for RT-qPCR were used as follows: IL-1β (ATTTGAGTCTGCCCAGTTCC and GTTATATCCTGGCCGCCTTT), IL-6 (GGAGACTTGCCTGGT GAAA and CTCTGGCTTGTTCCTCACTAC), IL-8 (CTCTGTG TGAAGGTGCAGTT and TTTCTGTGTTGGCGCAGT), CC L5 (AGATCTCTGCAGCTGCCCTCA and GGAGCACTTGCT GCTGGTGTAG), TNF-α (CCTCTCTCTAATCAGCCCTCT and TGCTACAACATGGGCTACAG), and GAPDH (CTCCTCC TGTTCGACAGTCAGC and CCCAATACGACCAAATCCGTT).

### Western blotting

Total proteins were extracted from HGFs using RIPA lysis buffer and separated by 10% SDS-PAGE. Subsequently, proteins were transferred to a 0.22 μm PVDF membrane (Millipore, United States) and blocked with 5% non-fat milk for 1 h at room temperature. The membranes were then incubated overnight at 4 °C with the following primary antibodies: anti-p16, anti-p21, and anti-β-actin (all from Proteintech, China). After washing, the membranes were probed with appropriate HRP-conjugated secondary antibodies for 1 h at room temperature. Protein bands were visualized using an enhanced chemiluminescence detection system (Bio-Rad, United States).

### Mouse periodontitis model and treatments

Male C57BL/6 mice (6–8 weeks old; 17–21 g) were obtained from the Animal Research Center of Xi’an Jiaotong University and maintained under specific pathogen-free conditions. All animal experiments were approved by the Animal Research Committee of Xi’an Jiaotong University (approval no. 2025–2642; 24 April 2025). A periodontitis model was established using a ligature-induced method. Briefly, mice were anesthetized with 4% isoflurane, and a 5–0 silk ligature was placed around both left and right maxillary first molar for 14 days. After ligature removal, the mice were randomly allocated into two groups (*n* = 6 per group): the PBS group and the 5-MTHF group. Intragingival injections were performed every 3 days, with each mouse receiving 10 μL of either sterile PBS or 5-MTHF solution at a dose of 15 μg/kg body weight. All animals were euthanized 30 days after the initiation of treatment for subsequent analysis.

### Micro-CT analysis

An X-ray microcomputed tomography (micro-CT) system (PerkinElmer, 25 mm FOV, 90 kV, 88 μA, 50 μm Voxel Size, 240 s exposure) was utilized to scan the mouse maxillae. The obtained data were reconstructed and analyzed with Mimics 21.0 (Materialize), and two-dimensional images were obtained using 3-matic Research 13.0 (Materialize) software. Alveolar bone loss (ABL) was assessed by measuring the distance from the cementoenamel junction (CEJ) to the alveolar bone crest (ABC) at the lingual and buccal regions of the first molar.

### Histological analyses

Mouse maxillae were fixed in 4% paraformaldehyde (PFA), decalcified in 10% EDTA, and subsequently dehydrated through a graded ethanol series. Tissues were then embedded in paraffin, and 5 μm-thick sections were cut. Sections were stained with hematoxylin and eosin (H&E) for histological examination.

### Statistical analysis

Weights were considered in this study following the NHANES analysis guide. The new 4-year weights were calculated using half of the 2-year MEC weights. Continuous variables were described as means and standard errors (SEs), and categorical variables were presented as percentages. Weighted multivariable regression models were employed to assess the link between serum 5-MTHF, biological aging and periodontitis. Model I was adjusted for age, sex and race. Model II was additionally adjusted for educational level, annual household income, smoking status, recreational activity, work activity, obesity, HEI-2015, diabetes mellitus, cardiovascular disease, and hypertension.

To investigate whether the relationship between serum 5-MTHF and periodontitis might be mediated by biological aging, we conducted an exploratory, hypothesis-generating mediation analysis using the “mediation” R package. Due to the cross-sectional design, temporal ordering cannot be established, and the results should be interpreted as correlational rather than causal. For continuous outcomes (mean AL and mean PPD), linear regression models were fitted, and the reported direct, indirect, and total effects represent unstandardized regression coefficients. For the binary outcome (periodontitis), logistic regression models were used, and the effects are reported as log-odds estimates. All mediation analyses employed non-parametric bootstrapping with 1,000 resampling iterations, and percentile 95% confidence intervals (CI) were calculated for the direct, indirect, and total effects. The direct effect (DE) was utilized to evaluate the impact of serum 5-MTHF on periodontitis in the absence of a mediator, while the indirect effect (IE) measured the influence of serum 5-MTHF on periodontitis through the mediator. The proportion of mediation was calculated by dividing the IE by the total effect (TE). A complete case analysis approach was performed in this study owing to the negligible amount of missing data.

For the *in vitro* experiments, we performed at least three independent biological replicates, each with three technical replicates. For micro-CT and histological analyses, six animals per group were used as biological replicates, with no technical replicates, and the experiment was performed once. All histological sections were evaluated by two independent investigators blinded to group allocation throughout the analysis. All quantitative data are presented as mean ± standard deviation (SD). Differences between two groups were compared using Student’s *t*-test, whereas comparisons across multiple groups were conducted using one-way analysis of variance (ANOVA). All statistical analyses were performed with R software (version 4.1.2; R Foundation for Statistical Computing, Vienna, Austria), and a *P*-value < 0.05 was considered statistically significant.

## Results

Supplementary Figure 1 details the participant screening workflow. Out of a total of 19,931 participants from the NHANES 2011–2014, an initial evaluation was conducted on 6,940 individuals who underwent comprehensive periodontal health examinations. After excluding subjects lacking data for 5-MTHF measurement or biological aging metrics (*n* = 695), the final analytical cohort comprised 6,245 participants, representing approximately 135.9 million non-institutionalized residents of the United States. Table 1 summarizes the baseline characteristics of the study participants, both overall and according to different serum 5-MTHF level categories. The mean age of the study was 50.98 years, and females comprised 50.52% of the total sample. Notably, the prevalence of moderate/severe periodontitis showed a progressive decreased with higher serum 5-MTHF levels, with rates of 45.13%, 37.84%, 33.58%, and 32.08% across the respective groups.

**TABLE 1:** Characteristics of the participants.

Characteristics	5-MTHF					*P*-value
	Overall	Q1	Q2	Q3	Q4	
Age (years), mean (SE)	50.98 (0.32)	47.82 (0.46)	48.80 (0.45)	49.95 (0.43)	56.52 (0.54)	<0.0001
Age group (%)						<0.0001
≤60	74.78	82.71	82.31	77.88	58.62	–
>60	25.22	17.29	17.69	22.12	41.38	–
KDM-BA, mean (SE)	−2.85 (0.24)	−0.44 (0.55)	−2.76 (0.43)	−3.51 (0.43)	−4.33 (0.44)	<0.0001
PhenoAge, mean (SE)	−0.52 (0.11)	0.53 (0.17)	−0.27 (0.15)	−0.68 (0.25)	−1.48 (0.17)	<0.0001
Sex (%)						<0.0001
Female	50.52	46.56	45.63	48.95	59.62	–
Male	49.48	53.44	54.37	51.05	40.38	–
Race (%)						<0.0001
Non-Hispanic White	69.28	62.71	63.5	69.78	79.38	–
Mexican American	8.09	8.18	10.34	9.46	4.74	–
Non-Hispanic Black	9.67	17.09	10.8	7.28	4.75	–
Others	12.96	12.02	15.36	13.47	11.14	
Education^a^ (%)						<0.001
<High school	13.77	17.98	14.5	12.4	10.94	–
High school	20.26	21.81	21.35	19.24	18.99	–
>High school	65.94	60.2	64.15	68.36	70.07	–
Marital status^a^ (%)						<0.001
Never married	10.36	14.64	10.76	9.8	6.98	–
Married/living as married	69.59	65.26	69.25	71.6	71.62	–
Separated/divorced/widowed	20.04	20.1	20	18.59	21.4	–
Income^a^ (%)						<0.001
<US$20,000	11.67	14.6	13.33	10.14	10.6	–
>US$20,000	85.25	85.4	86.67	89.86	89.4	–
Smoking status^a^ (%)						<0.0001
Non smoker	55.93	49.97	51.17	59.5	61.77	–
Current smoker	44.04	50.03	48.83	40.5	38.23	–
Recreational activity (%)						0.09
No	45.33	49.23	45.81	41.68	45.11	–
Moderate	31.08	28.58	31.49	32.07	31.89	–
Vigorous	7.06	7.58	7.41	7.11	6.27	–
Both	16.53	14.62	15.3	19.13	16.73	–
Work activity^a^ (%)						0.09
No	58.88	58.63	57.55	58.26	60.89	–
Moderate	21.44	19.59	20.32	22.55	22.93	–
Vigorous	4.59	5.79	4.93	4.14	3.74	–
Both	15.07	15.99	17.21	15.06	12.44	–
Obesity^a^ (%)						<0.001
No	61.79	56.16	60.98	63.89	67.47	–
Yes	37.23	43.84	39.02	36.11	32.53	–
Diabetes mellitus^a^ (%)						0.01
No	85.47	88.86	86.65	84.22	84.3	
Yes	14.04	11.14	13.35	15.78	15.7	
Hypertension (%)						0.002
No	58.95	60.64	58.92	63.04	53.68	–
Yes	41.05	39.36	41.08	36.96	46.32	–
Cardiovascular disease^a^ (%)						0.001
No	92.85	93.65	93.99	93.89	90.24	–
Yes	7.14	6.35	6.01	6.11	9.76	–
HEI-2015, mean (SE)	55.66 (0.31)	51.34 (0.48)	54.15 (0.63)	56.82 (0.53)	59.53 (0.55)	<0.0001
Mean AL, mean (SE)	1.68 (0.03)	1.83 (0.04)	1.73 (0.05)	1.59 (0.03)	1.59 (0.04)	<0.0001
Mean PPD, mean (SE)	1.36 (0.02)	1.50 (0.03)	1.40 (0.03)	1.32 (0.03)	1.24 (0.02)	<0.0001
Periodontitis (%)						<0.0001
No/mild	63.18	54.87	62.16	66.42	67.92	–
Moderate/severe	36.82	45.13	37.84	33.58	32.08	–

^a^Missing values for total study: education (*n* = 4, 0.06%), marital status (*n* = 3, 0.05%), income (*n* = 257, 4.12%), smoking status (*n* = 4, 0.06%), work activity (*n* = 2, 0.03%),obesity (*n* = 63, 1.00%), diabetes mellitus (*n* = 29; 0.46%), cardiovascular disease (*n* = 1, 0.02%), and HEI-2015 (*n* = 378, 6.05%).

**FIGURE 1:**
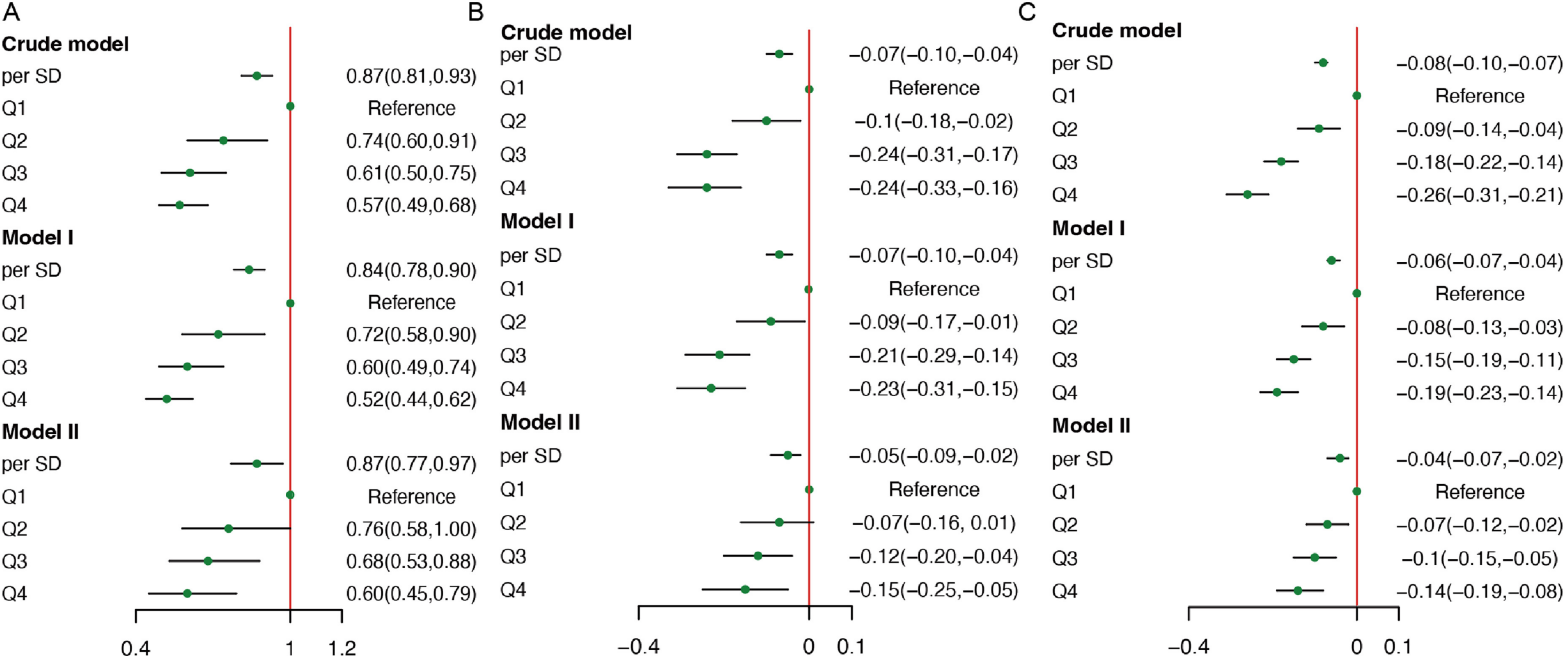
**(A)** Relationship between 5-methyltetrahydrofolate (5-MTHF) and periodontitis. **(B)** Relationship between 5-MTHF and mean attachment loss (AL). **(C)** Relationship between 5-MTHF and mean PPD.

The weighted regression analysis revealed a significant negative relationship between 5-MTHF, periodontitis, mean AL and mean PPD in the fully adjusted models (OR = 0.87, 95% CI: 0.77 to 0.97, β = −0.05, 95% CI: −0.09 to −0.02 and β = −0.04, 95% CI: −0.07 to −0.02, respectively) per SD increase in 5-MTHF levels. Similarly, 5-MTHF levels were grouped into quartiles also exhibited a negative correlation with periodontitis (OR_*Q4vs1*_ = 0.60, 95% CI: 0.45 to 0.79), mean AL (β_*Q4vs1*_ = −0.15, 95% CI: −0.25 to −0.05) and mean PPD (β_*Q4vs1*_ = −0.14, 95% CI: −0.19 to −0.08), respectively (Figures 1A–C).

**FIGURE 2:**
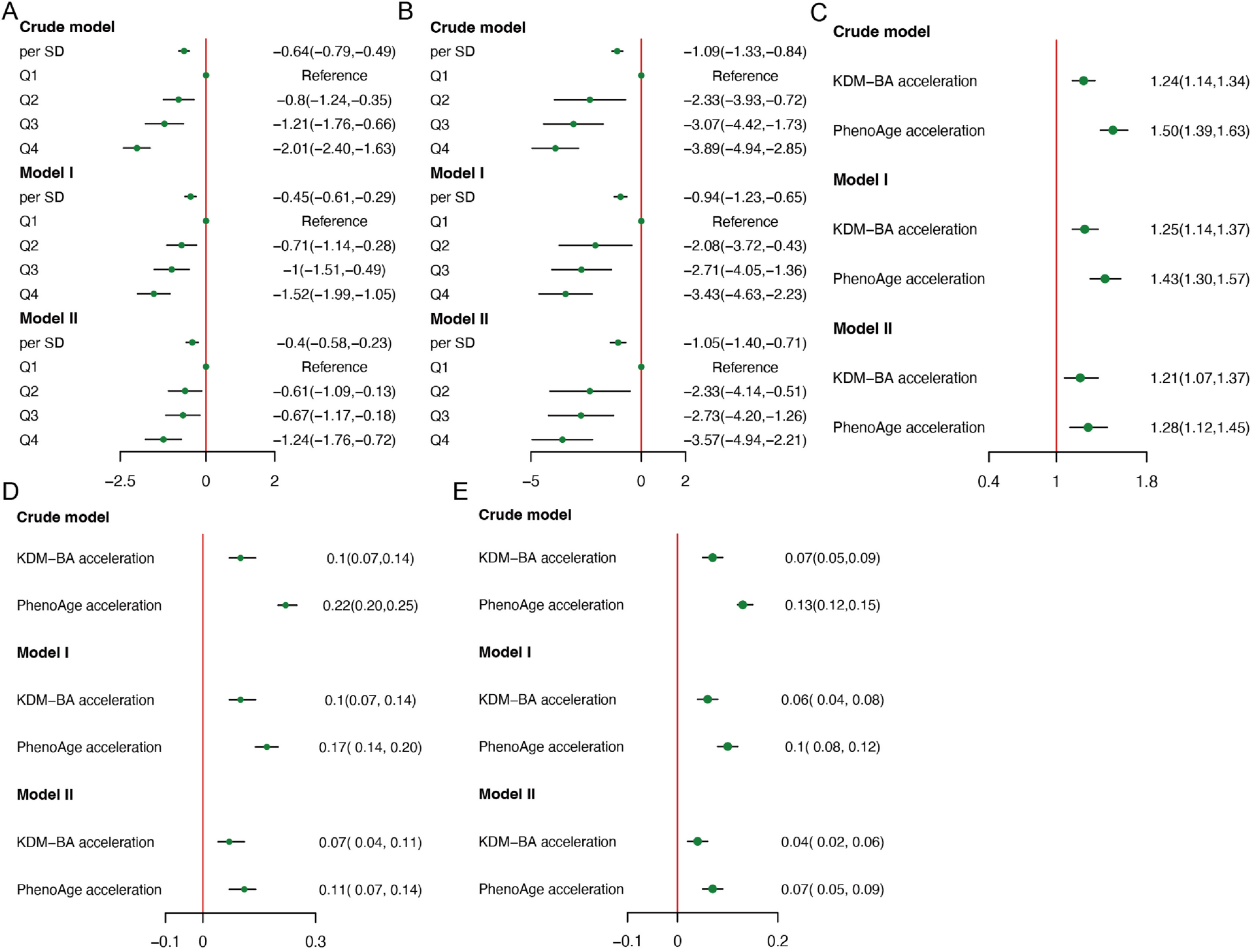
**(A)** Relationship between 5-methyltetrahydrofolate (5-MTHF) and Phenotypic Age (PhenoAge) acceleration. **(B)** Relationship between 5-MTHF and KDM-BA acceleration. **(C)** Relationship between PhenoAge acceleration, Klemera-Doubal method biological age (KDM-BA) acceleration, and periodontitis. **(D)** Relationship between PhenoAge acceleration, KDM-BA acceleration, and mean attachment loss (AL). **(E)** Relationship between PhenoAge acceleration, KDM-BA acceleration, and mean PPD.

**FIGURE 3:**
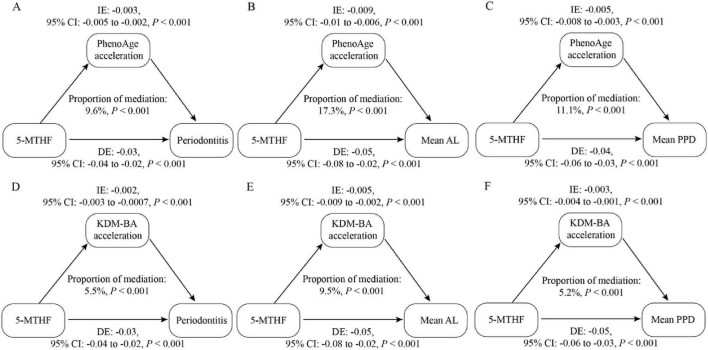
5-MTHF was associated with periodontitis **(A)**, mean AL **(B)**, mean PPD **(C)** through the PhenoAge acceleration. 5-MTHF was associated with periodontitis **(D)**, mean AL **(E)**, mean PPD **(F)** through the KDA-BA acceleration.

Further exploratory mediation analyses were performed to assess the potential role of biological aging in partially explaining the relationship between 5-MTHF and periodontal health. The findings revealed that 5-MTHF was associated with PhenoAge acceleration (β_*per SD*_ = −0.40, 95% CI: −0.58 to −0.23, β_*Q4vs1*_ = −1.24, 95% CI: −1.76 to −0.72) and KDM-BA acceleration (β_*per SD*_ = −1.05, 95% CI: −1.40 to −0.71, β_*Q4vs1*_ = −3.57, 95% CI: −4.94 to −2.21), respectively (Figures 2A, B). Additionally, the study indicated that KDM-BA acceleration and PhenoAge acceleration were both associated with increased risks of periodontitis (OR = 1.21, 95% CI: 1.07 to 1.37 and OR = 1.28, 95% CI: 1.12 to 1.45), mean AL (β = 0.07, 95% CI: 0.04 to 0.11 and β = 0.11, 95% CI: 0.07 to 0.14, respectively) and mean PPD (β = 0.04, 95% CI: 0.02 to 0.06 and β = 0.07, 95% CI: 0.05 to 0.09, respectively) (Figures 2C–E). Regarding mediation effects, PhenoAge acceleration was found to mediate 9.6%, 17.3%, and 11.1% of the associations between 5-MTHF, periodontitis, mean AL, and mean PPD, respectively. Similarly, KDM-BA acceleration mediated a larger proportion, accounting for 5.5%, 9.5%, and 5.2% of these relationships, respectively (Figure 3).

**FIGURE 4:**
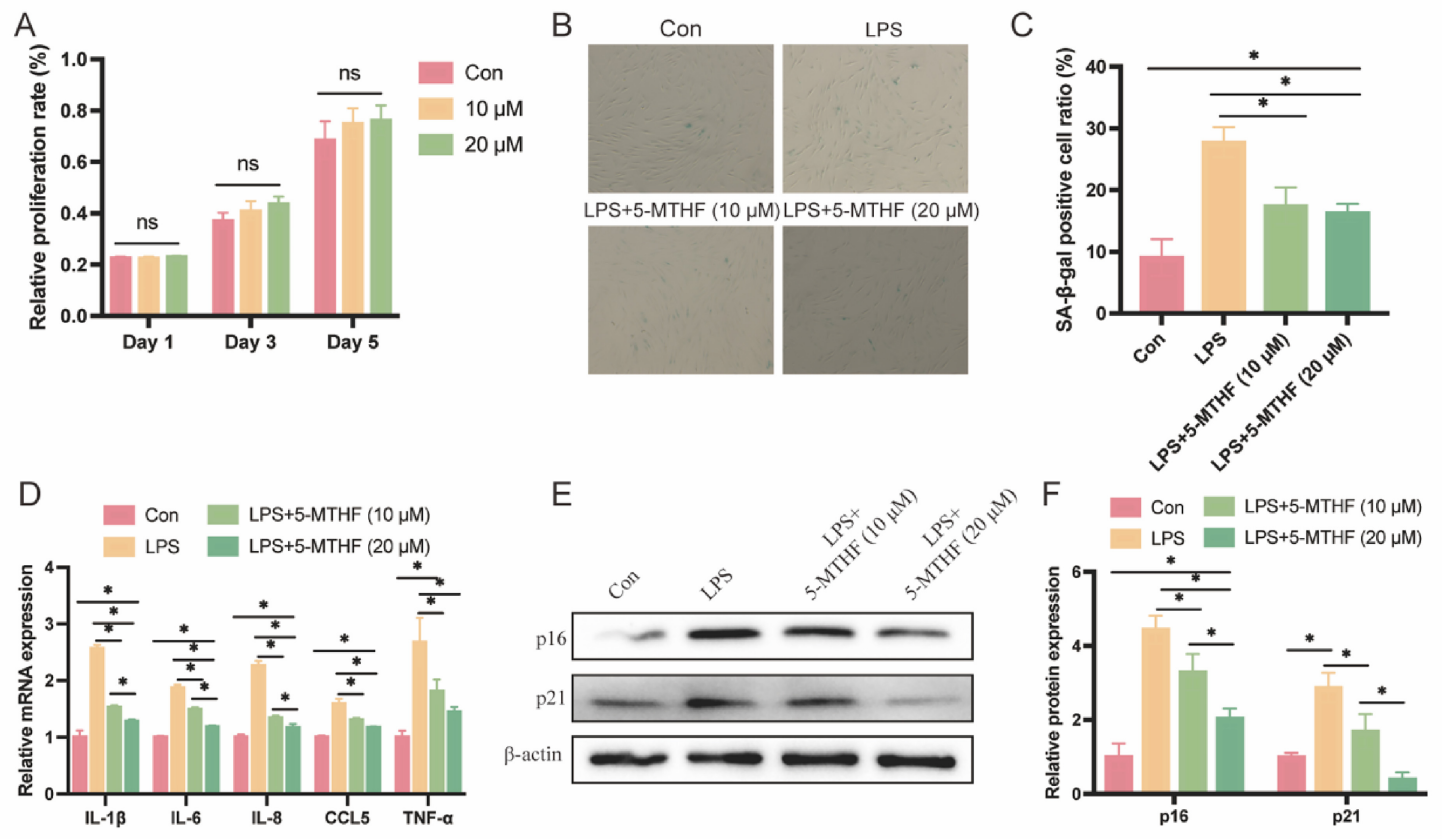
5-methyltetrahydrofolate (5-MTHF) attenuates lipopolysaccharide (LPS)-induced senescence in human gingival fibroblasts (HGFs) *in vitro.* **(A)** Cell Counting Kit-8 assay was conducted to evaluate the cytotoxic effects of 5-MTHF on HGFs. **(B)** Representative images of SA-β-gal staining. **(C)** Ratio of senescent cells was quantitatively analyzed. **(D)** mRNA expression levels of the pro-inflammatory cytokines IL-1β, IL-6, IL-8, CCL5, and TNF-α; **(E,F)** Protein expression of the senescence markers p16 and p21. *p < 0.05.

**FIGURE 5:**
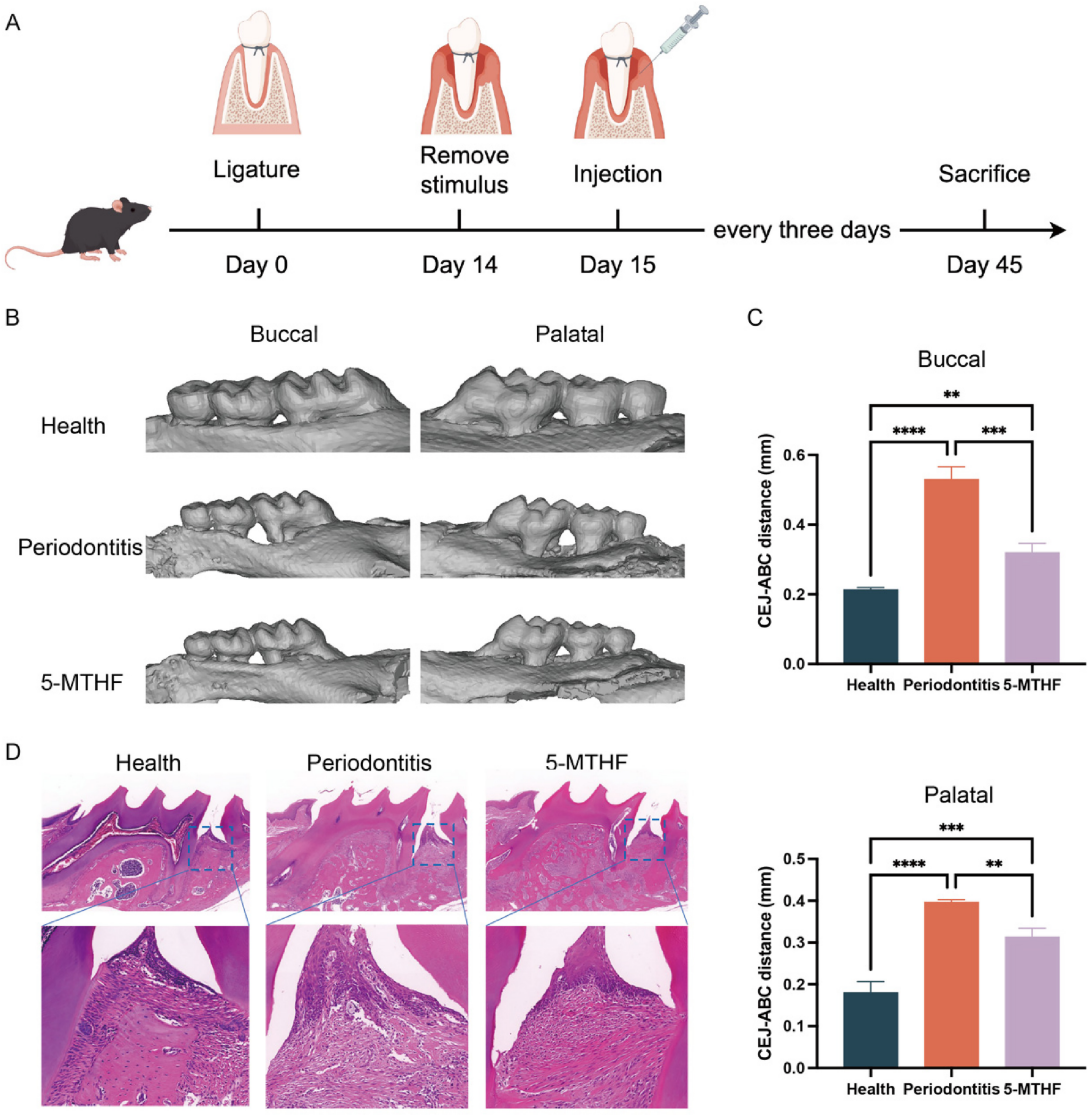
Therapeutic effect evaluation of 5-methyltetrahydrofolate (5-MTHF) *in vivo.* **(A)** Schematic illustration for ligature-induced periodontitis model and local administration of PBS and 5-MTHF by Figdraw. **(B)** 3D reconstruction of the maxillary molars by microcomputed tomography (micro-CT). **(C)** Statistical analysis of the CEJ-ABC distance. **(D)** Hematoxylin and eosin (H&E) staining images of periodontal tissue. **p < 0.01, ***p < 0.001.

To investigate the role of 5-MTHF in LPS-induced senescence of HGFs, we first evaluated the cytotoxicity of 5-MTHF. We found that 5-MTHF at 10 and 20 μM had no effect on HGFs proliferation (Figure 4A). We then treated HGFs with 5-MTHF and examined key senescence markers. Our results showed that compared with LPS treatment alone, co-treatment with 5-MTHF significantly alleviated cellular senescence, with a more prominent effect at 20 μM. This was evidenced by decreased SA-β-gal-positive cell ratio, reduced protein levels of p16 and p21, and downregulated mRNA expression of senescence-associated secretory phenotype (SASP) factors including IL-1β, IL-6, IL-8, CCL5 and TNF-α (Figures 4B–F). Moreover, *in vivo* experiments demonstrated that local injection of 5-MTHF markedly reduced alveolar bone loss compared to the control group (Figures 5A–C). Histopathological examination via H&E staining further indicated that the 5-MTHF group exhibited denser and more orderly arranged elastic and collagen fibers in the gingival tissues. This improved tissue architecture was associated with a substantial reduction in inflammatory cell infiltration, making it closely resemble that of normal gingival structure (Figure 5D).

## Discussion

In this study, our findings reveal a significant positive association between 5-MTHF levels and periodontal health status, with biological aging serving as a partial mediator. *In vitro* experiments established that 5-MTHF suppresses LPS-induced cellular senescence in HGFs. Subsequent *in vivo* investigations confirmed that local administration of 5-MTHF significantly reduces alveolar bone resorption in experimental periodontitis. These collective results position 5-MTHF as a promising therapeutic candidate for periodontitis treatment.

5-MTHF is the predominant circulating form of folate, accounting for 82%–93% of total blood folate (14, 15). Epidemiological evidence consistently links folate status with periodontal health. A large population-based study reported that older adults with higher serum folate levels had a 27% reduced risk of periodontitis (16). Furthermore, red blood cell (RBC) folate levels, indicative of long-term folate status, are inversely correlated with periodontitis severity (17). Clinical intervention trials corroborate these observations, indicating that systemic folic acid supplementation as an adjunct to scaling and root planning yields superior clinical outcomes, such as reduced gingival recession and attachment loss, compared to mechanical therapy alone (18). Notably, folic acid, a synthetic form used in supplements and fortified foods, must be converted to active forms like 5-MTHF (19). Complementing this clinical evidence, our *in vivo* investigations confirmed that local administration of 5-MTHF significantly attenuates alveolar bone resorption in experimental periodontitis. Together, this convergence of epidemiological, clinical, and experimental evidence underscores the protective role of 5-MTHF in periodontal health.

Although the biological mechanism underlying the association between 5-MTHF and periodontitis has not been fully elucidated, our findings point to a potential mediating role of aging, supported by exploratory mediation analyses and *in vitro* experimental evidence. As the bioactive coenzyme of one-carbon metabolism, 5-MTHF is integral to DNA synthesis, methylation, and redox homeostasis, processes closely associated with aging (20, 21). Furthermore, 5-MTHF appears to reduce levels of methylmalonic acid (MMA), a biomarker of mitochondrial dysfunction associated with inflammation, oxidative stress, and accelerated aging (22–24). Notably, our previous analysis of NHANES data revealed an inverse correlation between MMA and periodontal health, alongside a positive association between MMA and biological aging (2, 25). Collectively, these anti-inflammatory, antioxidant, and anti-aging properties provide an integrative framework for understanding 5-MTHF’s therapeutic potential in managing periodontitis, underscoring the need for further research to fully elucidate these pathways.

The present study’s findings hold significant importance for clinical research and carry notable implications for public health. Clinically, the elucidation of biological aging as a partial mediator and the direct evidence that 5-MTHF inhibits cellular senescence and alveolar bone resorption establish a strong mechanistic rationale for its therapeutic application. This positions 5-MTHF as a novel, pathogenesis-targeted agent that could be developed as an adjunctive therapy, potentially enhancing the outcomes of standard periodontal treatments, especially in aging populations or cases refractory to conventional care. From a public health perspective, the positive association between systemic 5-MTHF levels and periodontal health highlights the role of nutritional status, a modifiable risk factor, in a highly prevalent global disease. This insight advocates for the potential integration of folate status assessment and supplementation into broader preventive oral health strategies and community health programs. Given its safety, oral bioavailability, and relatively low cost, 5-MTHF represents a feasible and scalable intervention that could help reduce the population-wide burden of periodontitis and its associated systemic comorbidities, offering a cost-effective approach to improve oral and general health outcomes.

This study demonstrates several notable strengths. To our knowledge, this represents the first population-based investigation to identify biological aging as a mediator in the relationship between 5-MTHF and periodontitis, with the observed association remaining significant after adjusting for potential confounders. Moreover, *in vitro* experiments indicated that 5-MTHF inhibits LPS-induced senescence in HGFs, and *in vivo* studies further confirmed that 5-MTHF significantly attenuates alveolar bone resorption. However, several limitations warrant consideration. First, the cross-sectional design precludes causal inferences between 5-MTHF levels and periodontal health. Second, single-time-point serum sampling may not accurately reflect long-term 5-MTHF concentrations. Thirdly, residual confounding from unmeasured factors could not be fully eliminated. Fourth, our *in vitro* investigation focused exclusively on HGFs, which do not represent the full repertoire of immune cells involved in periodontitis pathogenesis. The absence of immune-cell analyses limits the interpretation of the anti-inflammatory effect at the multicellular level. Fifth, the lack of time-course data and an untreated control group in the *in vitro* experiments constrains the generalizability of our findings. Sixth, the *in vivo* data remain largely descriptive and lack mechanistic depth, including assessments of osteoclast activity (TRAP staining), senescence markers (p16/p21) and inflammatory cytokines (IL-1β, TNF-α, IL-6). Despite these limitations, our results provide initial evidence that 5-MTHF can attenuate LPS-induced inflammatory and senescence responses *in vitro* and reduce alveolar bone resorption *in vivo*. Future studies should incorporate longitudinal designs, repeated biomarker measurements, comprehensive confounding control, co-culture systems or *in vivo* models to evaluate immune-cell involvement, more complete experimental designs with time-course analyses, and systematic mechanistic validation of senescence markers. We have also made substantial progress in a follow-up project using a biomaterial-based delivery system for 5-MTHF, which will address several of these limitations and will be reported in a subsequent publication.

## Conclusion

In summary, our work provides evidence that 5-MTHF improves periodontal health partly through attenuating cellular senescence. This highlights the potential of targeting senescence via nutritional intervention with 5-MTHF as a promising therapeutic approach for periodontitis.

## Data Availability

Publicly available datasets were analyzed in this study. This data can be found here: https://wwwn.cdc.gov/nchs/nhanes/Default.aspx.
