# Supplementary material for: Targeting senescence with 5-methyltetrahydrofolate: a novel strategy to attenuate periodontitis
Source: Front Nutr. 2026 Aug 4;13:1851692. doi: 10.3389/fnut.2026.1851692 (PMC13481309; doi:10.3389/fnut.2026.1851692)
Supplement: Supplementary file 1 [file Table_1.docx]

*Table S1* The definition of periodontitis based on CDC/AAP

| Category | Definition |
| --- | --- |
| Healthy | No evidence of mild, moderate, or severe periodontitis |
| Mild periodontitis | ≥2 interproximal sites with AL ≥3 mm, and ≥2 interproximal sites with PD ≥4 mm (not on same tooth) or 1 site with PPD ≥5 mm |
| Moderate periodontitis | ≥2 interproximal sites with PPD ≥ 5 mm, not on the same tooth, or ≥2 interproximal sites with AL ≥ 4 mm, not on the same tooth |
| Severe periodontitis | ≥2 interproximal sites with AL ≥ 6 mm, not on the same tooth, and ≥1 interproximal site with PPD ≥ 5 mm |

**Figure S1.** Study population selection (N = 6,245) by Figdraw.
